# Intra-operative hypertension as a predictor of surgical outcomes in microvascular decompression surgery for trigeminal neuralgia

**DOI:** 10.1007/s00701-024-06178-9

**Published:** 2024-07-15

**Authors:** Bhavika Gupta, Mohammadmahdi Sabahi, Romel Corecha Santos, Yatin Srinivash Ramesh Babu, Raphael Augusto Correa Bastianon Santiago, Rocco Dabecco, Simone Phang-Lyn, Badih Adada, Hamid Borghei-Razavi

**Affiliations:** 1 Department of Neurological Surgery, Pauline Braathen Neurological Center, Cleveland Clinic Florida, Weston, FL USA; 2 Department of Neurosurgery, Allegheny General Hospital, Pittsburgh, PA USA; 3 Dr. Kiran C. Patel College of Osteopathic Medicine, Nova Southeastern University, Fort Lauderdale, FL USA; 4 Department of Anesthesiology, Cleveland Clinic Florida, Weston, FL USA

**Keywords:** Trigeminal nerve, Hypertension, Trigeminocardiac reflex, Microvascular decompression, Pain

## Abstract

**Purpose:**

The trigeminocardiac reflex (TCR) has traditionally been characterized by a sudden decrease in heart rate, asystole, or hypotension during the manipulation of the trigeminal nerve (MTN) or its branches. While this classical TCR is well-documented, there is limited literature on alternative forms of TCR, such as the development of intraoperative hypertension (HTN) or tachycardia, and the underlying pathogenesis. Furthermore, a gap exists in understanding the correlation between intraoperative blood pressure readings and postoperative outcomes, particularly regarding pain relief in patients with trigeminal neuralgia (TN). Our study aims to examine intraoperative blood pressure trends during microvascular decompression (MVD) for TN and assess their impact on postoperative outcomes.

**Methods:**

We selected 90 patients who underwent MVD for TN treatment. Blood pressure and heart rate were recorded both preoperatively and during the procedure, specifically during the MTN period, using an arterial line. The Barrow Neurological Institute (BNI) Pain Scale was calculated for all patients both pre- and post-operatively to evaluate pain relief after surgery.

**Results:**

The mean age of the patients was 61.0 ± 12.35 years, with 64.4% being females. Classical TCR (hypotension) was observed in only 2.2% of patients, whereas 80% of patients developed hypertension (≥ 140/90) during MTN. The mean preoperative systolic blood pressure was 128 ± 22.25, and the mean intraoperative systolic blood pressure during MTN was 153.1 ± 20.2. An analysis of covariance, utilizing either preoperative BNI or duration of symptoms as covariate variables, revealed a statistically significant association between intraoperative HTN and postoperative BNI. A linear regression model demonstrated that intraoperative HTN following MTN significantly predicted a lower postoperative BNI score (*p* = 0.006).

**Conclusions:**

Intraoperative HTN during MTN, an observed yet underexplored phenomenon, demonstrated a correlation with improved postoperative outcomes. Furthermore, it is recommended to conduct additional investigations into potential neurovascular conflicts in patients not manifesting intraoperative HTN following MTN. A comprehensive understanding of TCR, encompassing its various forms, is vital for optimizing surgical management. This study underscores the imperative for further research to unravel the mechanisms linking intraoperative HTN to surgical outcomes in TN patients.

## Introduction

Trigeminal Neuralgia (TN) is a facial pain disorder and is characterized by sudden, severe episodes of facial pain that typically last for a few seconds. The pain is usually triggered by normal activities like speaking or eating [10]. The most common etiology for TN is believed to be related to compression or irritation of the trigeminal nerve by the surrounding vasculature [8]. Microvascular Decompression (MVD) is the gold standard surgical treatment for medically refractory classic trigeminal neuralgia [1]. The Trigeminal Cardiac Reflex (TCR) is a complex reflex involving the trigeminal nerve and the autonomic nervous system and is often triggered by stimulation of the trigeminal nerve, which sends signals to the brainstem and the cardiovascular centers, resulting in changes in heart rate and blood pressure [5]. When the trigeminal nerve is activated, it sends a signal to the brain which can result in several different outcomes. Any sensory stimulation of the nerve leads to activation of the Gasserian ganglion, which activates the reticular formation and in turn the cardioinhibitory parasympathetic vagal neurons. The sensory nucleus of the trigeminal neve is connected to the reticular formation via polysynaptic connections mediated endogenously by cholinergic and serotonin receptors [9]. Furthermore, the central circuit reflex lies within the brainstem which further mediates cardiac responses. While these underlying pathways describe the pathogenesis of TCR, the clinical manifestation, from mild reflexive response to severe life-threatening bradycardia, varies significantly. There are several forms of TCR, including excitatory and inhibitory reflexes. Classical TCR is defined as hypotension, bradycardia, and loss of consciousness, during manipulation of the trigeminal nerve (MTN) or its branches and is observed in up to 18% of MVDs performed for TN [22]. The TCR can also be modulated by other reflexes, such as the baroreceptor reflex [19]. Additionally, the TCR can be influenced by various factors, such as age, stress, and underlying disease. Understanding the role of the TCR is important for the management of various cardiovascular risk factors such as perioperative myocardial infarction and acute kidney injury during MVD [13]. While TCR is a protective physiological reflex, the exaggeration of responses can be deemed pathological. Understanding the mechanisms behind TCR, and the different outcomes it can cause is essential for the effective management of patients with certain medical conditions, such as trigeminal neuralgia. The appearance of TCR is widely reported in the literature [19, 21, 22], there are no reports on the different patterns of TCR observed during microvascular decompression surgery. Additionally, there is a paucity of literature on how developing TCR during surgery correlates with outcomes. To our knowledge, this is the first study of its kind to report on the surgical outcomes and post-surgical effects of developing intraoperative hypertension (HTN) during microvascular decompression surgery.

## Material and methods

### Data collection

We conducted a retrospective study with 90 patients who underwent MVD for medically refractory TN at our center from January 2013 to January 2023. Patient demographics such as age, sex, prior history of HTN, comorbidities, medications, involved trigeminal nerve branches, pre-operative vital signs, and intra-operative vital signs were collected via manual chart review. Changes in blood pressure and heart rate were recorded pre-operatively and during the procedure, specifically at the time of MTN. Both an arterial line, as well as a non-invasive blood pressure cuff, were used. This data was collected by reviewing the anesthesiologist’s intra-operative report. Additionally, the occurrence of TCR, if any, was recorded by the anesthesiologist at the time of the MTN. Additionally, the pre-and post-operative Barrow Neurological Index (BNI) pain scores for trigeminal neuralgia were calculated for all patients and used as the primary outcome measure for post-operative symptom relief.

### Statistical analysis

SPSS 29.0 statistical software (IBM Corp., Armonk, NY) was used for data analysis. Means and standard deviation were computed between the two groups with a Mann Whitney test for nonparametric continuous data, and with Chi square or Fisher exact test when appropriate for categorical data. Analysis of covariance (ANCOVA) and linear regression were performed. We checked the assumptions of ANCOVA, including homogeneity of regression (HOR) slopes, homogeneity of variances, and normality of residuals. None of the assumptions were violated. A *p*-value of 0.05 or less was defined as statistically significant.

## Results

### Patient characteristics

Our study included a total of 90 patients. Table 1 shows the demographic and clinical data of the patients. The mean age of the population was 61.0 ± 12.35 years and included 32 (35.6%) males and 58 (64.4%) females. 35 patients (38%) had a prior history of HTN, and 25 patients (28%) had high blood pressure just prior to surgery. As documented by the anesthesiologist, only 2 (2.2%) patients developed the classical TCR (hypotension) whereas 88 (80%) patients experienced the hypertensive variant of TCR (BP > 140/90) during MTN.

**Table 1:** Comparison of demographic and clinical features between the two groups in the study population

	Intra-operative HTN (*n* = 72)	No Intra-operative HTN (*n* = 18)	*p*-value
Sex			0.378
Male Female	24 (33.3%) 48 (66.7%)	8 (8.6%) 10 (10.8%)	
Age^#^	65 years (26–83 years)	60 years (34–74 years)	0.073
PMH of HTN	30 (41.7%)	5 (5.4%)	0.280
PMH of DM	9 (23.6%)	1 (1.1%)	0.680
Obesity	17 (23.6%)	7 (7.5%)	0.235
Smoking	33 (45.8%)	9 (9.7%)	0.751
Type of Compression			0.210
Arterial Venous Mixed Nerve	49 (68.1%) 14 (19.4%) 5 (6.9%) 4 (5.6%)	11 (7.5%) 3 (3.2%) 4 (4.3%) 0	
Symptom Laterality			0.745
Right Left	38 (52.8%) 33 (45.8%)	11 (11.8%) 7 (7.5%)	
Branch			0.231
V1 V2 V3 Mixed	1 (1.4%) 24 (33.3%) 20 (27.8%) 27 (37.5)	1 (1.1%) 2 (2.2%) 3 (6%) 9 (9.7%)	
Pre-operative HTN	21 (29.2%)	4 (4.3%)	0.556
Pre-operative BNI^#^	5 (3–5)	4 (3–5)	0.128
Post-operative BNI^#^	1 (1–4)	2 (1–4)	**0.006**
Length of symptoms ^#^	36 days (1–360 days)	36 days (2–240 days)	0.266

Boldface type indicates statistical significance (*p* < 0.05)

*BNI* barrow neurological institute pain scale, *HTN* hypertension, *PMH* past medical history, *DM* diabetes mellitus

^#^Continuous data is presented as median (Range)

**Fig. 1:**
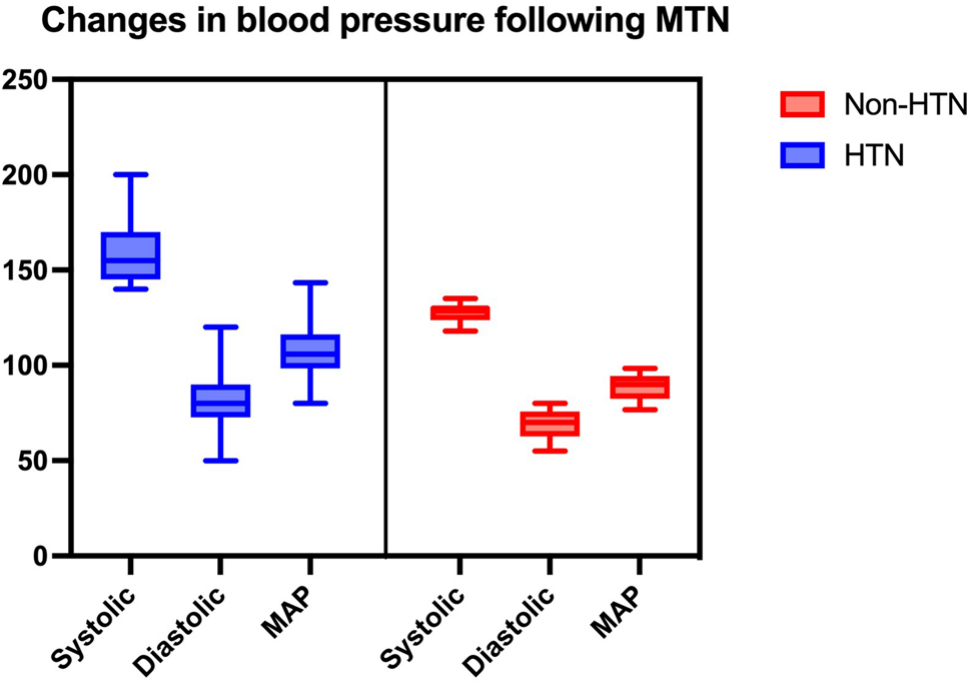
Mean arterial pressure (MAP), systolic, and diastolic blood pressure following manipulation of trigeminal nerve (MTN) in both groups. HTN: hypertension

**Fig. 2:**
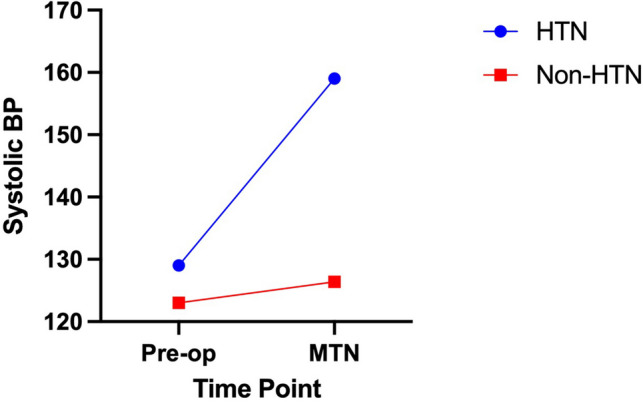
Blood pressure variations from pre-operative to intra-operative assessment during manipulation of trigeminal nerve (MTN) for both study groups. HTN: hypertension, BP: blood pressure

The mean pre-operative systolic BP was 128 ± 22.24 mmHg, and the mean intraoperative systolic BP was 153.1 ± 20.2 mmHg. Mean arterial pressure, systolic and diastolic BP following MTN in both groups has been demonstrated in Fig. 1. Minimum and maximum intra-operative pulse recordings were 60 ± 12.27 beat per minute (BPM) and 78.15 ± 12.67 BPM, respectively. Changes in BP from pre-operative assessment to intra-operative assessment during MTN have been illustrated in Fig. 2 for both groups.

### Outcome evaluation

**Fig. 3:**
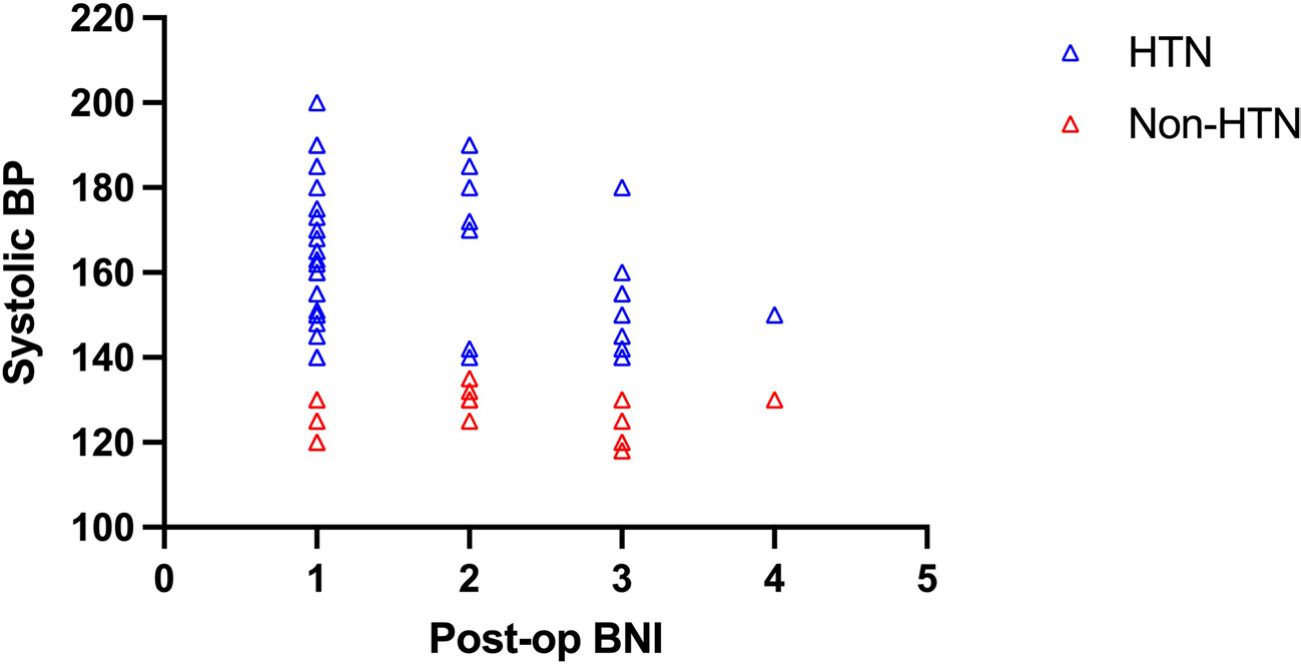
Distribution of systolic blood pressure in both groups, categorized according to post-operative Barrow Neurological Institute (BNI) pain scores. HTN: hypertension, BP: blood pressure

The distribution of systolic blood pressure in both groups, categorized according to post-operative BNI, is depicted in Fig. 3. The main aim of the analysis was to study the relationship between developing intra-operative HTN and post-operative BNI score, while controlling for the pre-operative BNI score and duration of symptoms. Some other factors that are thought to affect outcomes of MVD such as laterality of symptoms, branches of trigeminal nerve involved, etiology of nerve compression and duration of symptoms prior to surgery were also analyzed [4, 7, 15]. Additional univariate analyses of covariance were performed to study the effect of demographic variables such as age, sex, history of HTN, history of diabetes, smoking status, and obesity on post operative BNI scores.

For the analysis, patients were divided into two groups. The first group did not develop intra-operative HTN during MTN, while the second group developed sustained HTN during MTN for few minutes. Initially, we performed a univariate ANCOVA between intraoperative HTN and pre-operative BNI. The results showed no significant difference between the two groups regarding the BNI, thus allowing us to choose the pre-operative BNI as a covariate. Next, we performed HOR, which did not show any significant relationship either. After satisfying both these prerequisites an analysis of covariance performed. This process was repeated for duration of symptoms as the covariate, with post-operative BNI as the outcome.

In the first model with preoperative BNI as the covariate variable there was a statistically significant (*p* = 0.021) association between intraoperative HTN and postoperative BNI. A linear regression model for the same showed that patients who experienced intra-operative HTN have a 0.5-point decrease in post operative BNI as compared to patients who do not have intra-operative HTN, thus predicting that intraoperative HTN is associated with a lower postoperative BNI score.

The second model with duration of symptoms as the covariate, also showed a significant association (*p* = 0.007) between intra-operative HTN and post-operative BNI. Similarly, linear regression showed that patients who experienced intra-operative HTN have a 0.6-point decrease in post operative BNI as compared to patients who do not have intra-operative HTN, thus predicting that intraoperative HTN is associated with a lower postoperative BNI score, when controlling for duration of symptoms.

Additional univariate analyses demonstrated a significant relationship between past medical history of HTN and post operative BNI (*p* = 0.040), while controlling for pre-operative BNI. Further analyses of covariance with pre-operative BNI and duration of symptoms as covariates did not show any significant relationship between age, sex, pre-operative HTN, past medical history of diabetes, obesity, smoking status, laterality of symptoms, branches involved, etiology of compression and duration of symptoms with the post-operative BNI (Table 2).

**Table 2:** Relationship between patient factors and post-operative BNI

Fixed factor	*p*-value	
	*Pre-operative BNI	*Duration of symptoms
Intra-operative HTN	**0.021**	**0.007**
Sex	0.815	0.695
PMH of HTN	**0.040**	0.066
Immediate pre-operative HTN	0.865	0.862
Laterality	0.528	0.581
Branch	0.821	0.768
PMH of DM	0.275	0.361
Obesity	0.622	0.568
Smoking	0.685	0.472
Type of compression	0.507	0.518
Age	0.332	0.368
Duration of symptoms	0.252	−

Boldface type indicates statistical significance (*p* < 0.05)

*BNI* barrow neurological institute pain scale, *HTN* hypertension, *PMH* past medical history, *DM* diabetes mellitus

^*^Covariates in each individual univariate model

## Discussion

In our study of 90 patients, 72 (80%) developed episodes of HTN upon MTN during MVD. These patients had statistically significantly lower post-operative Barrow Neurological Index (BNI) scores and theoretically better outcomes post decompression, even after controlling for pre-operative BNI and duration of symptoms. Additionally, patients with a past medical history of HTN also had lower post-operative BNI scores when compared to patients who did not. The mean post-operative BNI scores was 1.46 for the group that developed intra-operative HTN as opposed to 2 for the group that did not develop HTN during the surgery.

The classical TCR in humans was first described by Schaller et al. in 1999 during cerebellopontine angle surgery [21]. Since then, he has defined TCR as a drop in mean arterial blood pressure by more than 20% upon stimulation of the trigeminal nerve complex [21]. To understand the different ways in which the TCR may present, it is important to understand the anatomy and the different trigger points available for the activation of the reflex.

The afferent limb of the TCR consists of sensory nerve fibers traveling to the Gasserian ganglion in Meckel's cave. From there, the afferent stimulus is transmitted to the sensory nucleus of the trigeminal nerve via the nerve's central part. Finally, internuncial fibers in the reticular formation connect the sensory nucleus of the trigeminal nerve with the nucleus ambiguous and the dorsal motor nucleus of the Vagus nerve, resulting in the TCR, which manifests clinically [6, 20].

Schaller et al. have further subdivided this reflex into three categories, depending on the anatomical location. The reflex includes the Central, Peripheral, and Ganglion subtype [21]. The peripheral reflex is triggered by a mechanical or chemical stimulus in the branches of the fifth cranial nerve, occurring after the Gasserian ganglion. In the ganglion subtype, the stimulus directly affects the Gasserian ganglion. The central/proximal TCR is observed when the trigger occurs in the intracranial part of the trigeminal nerve [19]. Both peripheral and central reflexes typically present with a decrease in heart rate. The central reflex is also associated with a decrease in blood pressure, which is not commonly seen in the peripheral reflex. In contrast, the "Gasserian ganglion" subtype can result in either an increase or decrease in heart rate and blood pressure [19]. Therefore, the afferent limb changes depending on the subtype of TCR (central, peripheral, or ganglion) [18, 19, 21].

Naturally, the most common effect of activating this response would be a decrease in blood pressure or heart rate, as is seen in classical TCR [18, 20, 21]. Review of the literature demonstrates the effects of TCR on both the parasympathetic (PNS) and sympathetic nervous system (SNS). Chen et al. showed that pre-treating with labetalol or anti-cholinergic agents prevented bradycardia and hypertension during balloon compression rhizotomy (BCR) [3]. Studies in animal models show that MTN can also lead to an increase in adrenaline release, thus activating the SNS.

Additionally, HTN during MTN has also been described in radiofrequency thermocoagulation (RFT). Foramen ovale puncture led to an increase in BP and heart rate (HR) in all the patients in a study done by Meng et al. Thermal energy was considerably stronger than electrical energy for causing increases in BP and HR and was found to be directly proportional to the amount of current that is directed at the lesioned site [17]. As the frequency of the thermal current increased, the depressor response converted to a stressor response. Additionally, the electrical stimulation and heating during RFT also heated up the C fibers leading to rises in the BP and pulse [17]. Additionally, if a patient has lighter anesthesia, the pain of the operation can lead to a severe sympathetic nervous response. Direct stimulation of the trigeminal ganglion may lead to a vasoconstrictive response, thus leading to HTN [12].

As shown by Schaller et al. the TCR has three components and, depending on the component stimulated, may have a different physiologic response [20]. Studies have demonstrated that the peripheral variant of the TCR has bradycardia, HTN and bouts of apnea. Whereas a more central manipulation leads to hypotension. Thus, depending on the area and degree of compression, MVD for TN may produce a different type of TCR [9, 16, 18, 20, 21]. Since the neurons within the ganglion are pseudo-unipolar and exhibit two axonal branches, the action potential can travel in either direction: orthodromically, moving from the Gasserian ganglion to the brainstem, or antidromically, moving toward the trigeminal branches [11]. Therefore, we hypothesize that during MVD, MTN stimulation leads to sensory input to the Gasserian ganglion, potentially explaining its activation. The Gasserian ganglion is surrounded by sympathetic and parasympathetic nerve fibers originating from the carotid plexus. This anatomic relationship may explain the variations in the TCR [2]. While developing the hypertensive variant of the TCR during MVD is not well studied, it is commonly observed and well-reported during other procedures involving MTN. Additionally, the balance of which the autonomic nervous system gets activated could vary from patient to patient making the response variable and unreliable.

A study performed by Liu et al. showed that more than 80% of patients experienced hypertension, during trigeminal nerve combing [14]. Additionally, these patients had an increase in the level of epinephrine, inferring that the increase in BP was directly related to the activation of the sympathetic nervous system. The authors concluded that the increased HTN was a result of vasoconstriction secondary to the sympathetic nervous system. Most interestingly, Patients who did not develop HTN during combing did not benefit from the procedure. They hypothesized, that the TN in these patients was likely due to a central complex, rather than peripheral compression syndrome. Further support for this theory was strengthened by the underlying diagnosis of multiple sclerosis (MS) in these sub-set of patients. Thus, these patients would not have benefited from the MVD. The major takeaway from their study was that it is important to consider this factor and be prepared for this occurrence, as such high increases in blood pressure can lead to severe intraoperative morbidity. Finally, one must be careful to avoid intracerebral hemorrhage, during MVD with such high blood pressures [14]. Like our results of improved outcomes in patients with blood pressure spikes, Zuo et al. reported that developing intra-operative HTN during BCR meant that the compression was successful, and further supported the use of continuous intra-operative blood pressure monitoring [23].

While most surgeons believe that it is best to prevent TCR rather than treat it intra-operatively, due to its various forms, pre-treatment with atropine is not recommended. However, there are limiting certain risk factors that can decrease the incidence of TCR. The best strategy is to prevent these risk factors and thus prevent the TCR from occurring [6]. In addition, the anesthesiology team should be alerted when the neurosurgeon is MTN and the neurosurgeon must always use gentle retraction while doing so. Finally, acknowledging the prognostic significance of intraoperative HTN subsequent to MTN, it is recommended to explore alternative causes of TN in patients who do not manifest this response. This ensures a thorough investigation, confirming that the surgeon has considered all potential sites for decompression in the treatment of trigeminal nerve.

## Limitations

The major limitation of this study is the small sample size and retrospective nature of this study. While our results were statistically significant, to deme their clinical significance and influence on surgical decision-making, a larger sample size and prospective studies must be conducted to determine the true effect of developing intra-operative HTN on outcomes of the procedure.

## Conclusion

As previously believed, the TCR exists in many forms. It is important to know the different anatomical locations of the trigeminal complex to understand what variant of the reflex will be evoked during surgery. The development of intra-operative HTN during MTN, regardless of either pre-operative BNI pain scale or duration of symptoms, may lead to better post-operative BNI scores and outcomes. As a result, the occurrence of intraoperative HTN subsequent to MTN may function as a prognostic indicator for surgical outcomes in patients with TN. Furthermore, it is recommended to conduct additional investigations into potential neurovascular conflicts in patients not manifesting intraoperative HTN following MTN. Further research is warranted to investigate the mechanisms underlying this association.

## Data Availability

The authors confirm that the data supporting the findings of this study are available within the article.

## References

[1] Allam AK, Sharma H, Larkin MB, Viswanathan A (2023) Trigeminal neuralgia: diagnosis and treatment. Neurol Clin 41:107–121 36400550 10.1016/j.ncl.2022.09.001

[2] Borghei-Razavi H, Das P, Maurtua M, Recinos PF (2018) Unusual appearance of trigemino-cardiac reflex during cerebellopontine angle surgery. World Neurosurgery 112:298–299 29580017 10.1016/j.wneu.2017.10.139

[3] Chen C-Y, Luo C-F, Hsu Y-C, Chen J-F, Day Y-J (2012) Comparison of the effects of atropine and labetalol on trigeminocardiac reflex-induced hemodynamic alterations during percutaneous microballoon compression of the trigeminal ganglion. Acta Anaesthesiol Taiwan 50:153–158 23385037 10.1016/j.aat.2012.11.001

[4] Cheng J, Meng J, Liu W, Zhang H, Hui X, Lei D (2017) Nerve atrophy in trigeminal neuralgia due to neurovascular compression and its association with surgical outcomes after microvascular decompression. Acta Neurochir 159:1699–1705 28638946 10.1007/s00701-017-3250-9

[5] Chowdhury T, Cappellani RB, Schaller B (2014) Chronic trigemino-cardiac reflex in patient with orbital floor fracture: role of surgery and first description. J Neurosurg Anesthesiol 26:91–92 23887683 10.1097/ANA.0b013e3182a1a691

[6] Chowdhury T, Mendelowith D, Golanov E, Spiriev T, Arasho B, Sandu N, Sadr-Eshkevari P, Meuwly C, Schaller B (2015) Trigeminocardiac reflex: the current clinical and physiological knowledge. J Neurosurg Anesthesiol 27:136–147 25602626 10.1097/ANA.0000000000000065

[7] Duan Y, Sweet J, Munyon C, Miller J (2015) Degree of distal trigeminal nerve atrophy predicts outcome after microvascular decompression for Type 1a trigeminal neuralgia. J Neurosurg 123:1512–1518 26186027 10.3171/2014.12.JNS142086

[8] Esmaeilzadeh M, Sabahi M, Maroufi SF, Dabeco R, Adada B, Roser F, Borghei-Razavi H (2023) When the nerve keeps firing: an institutional experience and systematic review on delayed response after microvascular decompression for trigeminal neuralgia. Neurol Sci 45(1):109–118 10.1007/s10072-023-07019-w 37676372

[9] Gorini C, Philbin K, Bateman R, Mendelowitz D (2010) Endogenous inhibition of the trigeminally evoked neurotransmission to cardiac vagal neurons by muscarinic acetylcholine receptors. J Neurophysiol 104:1841–1848 20719927 10.1152/jn.00442.2010 PMC2957459

[10] Green A, Nandi D, Armstrong G, Carter H, Aziz T (2003) Post-herpetic trigeminal neuralgia treated with deep brain stimulation. J Clin Neurosci 10:512–514 12852900 10.1016/s0967-5868(03)00088-2

[11] Huff T, Weisbrod LJ, Daly DT (2024) Neuroanatomy, Cranial Nerve 5 (Trigeminal). In: StatPearls. StatPearls Publishing 29489263

[12] Kehler CH, Brodsky JB, Samuels SI, Britt RH, Silverberg GD (1982) Blood pressure response during percutaneous rhizotomy for trigeminal neuralgia. Neurosurgery 10:200–202 7070616

[13] Kouz K, Hoppe P, Briesenick L, Saugel B (2020) Intraoperative hypotension: Pathophysiology, clinical relevance, and therapeutic approaches. Indian J Anaesth 64:90 32139925 10.4103/ija.IJA_939_19 PMC7017666

[14] Liu J, Wu G, Jiang Y, Li L, Wang D, Liu R (2020) Relationship between arterial blood pressure during trigeminal nerve combing and surgical outcome in patients with trigeminal neuralgia. World Neurosurg 137:e98–e105 31954896 10.1016/j.wneu.2020.01.038

[15] Loayza R, Wikström J, Grabowska A, Semnic R, Ericson H, Abu Hamdeh S (2023) Outcome after microvascular decompression for trigeminal neuralgia in a single center—relation to sex and severity of neurovascular conflict. Acta Neurochirurgica 165(7):1955–1962 37284837 10.1007/s00701-023-05642-2 PMC10319688

[16] McCulloch PF, Faber KM, Panneton WM (1999) Electrical stimulation of the anterior ethmoidal nerve produces the diving response. Brain Res 830:24–31 10350556 10.1016/s0006-8993(99)01374-8

[17] Meng Q, Zhang W, Yang Y, Zhou M, Li X (2008) Cardiovascular responses during percutaneous radiofrequency thermocoagulation therapy in primary trigeminal neuralgia. J Neurosurg Anesthesiol 20:131–135 18362775 10.1097/ANA.0b013e3181628305

[18] Meuwly C, Chowdhury T, Sandu N, Golanov E, Erne P, Rosemann T, Schaller B (2017) Definition and diagnosis of the trigeminocardiac reflex: a grounded theory approach for an update. Front Neurol 8:533 29085328 10.3389/fneur.2017.00533 PMC5649131

[19] Meuwly C, Golanov E, Chowdhury T, Erne P, Schaller B (2015) Trigeminal cardiac reflex: new thinking model about the definition based on a literature review. Medicine (Baltimore) 94(5):e484 10.1097/MD.0000000000000484 PMC4602726 25654391

[20] Schaller B (2004) Trigeminocardiac reflex: a clinical phenomenon or a new physiological entity? J Neurol 251:658–665 15311339 10.1007/s00415-004-0458-4

[21] Schaller B (2005) Trigemino-cardiac reflex during microvascular trigeminal decompression in cases of trigeminal neuralgia. J Neurosurg Anesthesiol 17:45–48 15632542

[22] Shibao S, Kenawy K, Borghei-Razavi H, Yoshida K (2017) The trigeminocardiac reflex during the anterior transpetrosal approach. World Neurosurg 106:939–944 28739515 10.1016/j.wneu.2017.07.086

[23] Zuo Y, Song D, Hu Y, Zhao S, Zhang M, Wang M, Guo F (2022) Continuous Intra-Arterial Blood Pressure Monitoring Improves the Efficiency of Percutaneous Balloon Compression of the Trigeminal Ganglion for Trigeminal Neuralgia. Pain Res Manag 3;2022:7567630 10.1155/2022/7567630 PMC9550456 36225719

